# Systematic analysis of the influence of enzymatic and chemical detergents on structure, biomechanics and biocompatibility of decellularized vascular grafts

**DOI:** 10.1007/s10856-025-06967-3

**Published:** 2025-11-26

**Authors:** Julian Pfarr, Karina Zitta, Georg Lutter, Sanjay Tiwari, Farhad Haj Mohamad, Philipp Knueppel, Frank Lichte, Katharina Hess, Mark Preuss, Sebastian Debus, Martin Albrecht, Rouven Berndt

**Affiliations:** 1 Department of Diagnostic and Interventional Radiology and Nuclear Medicine, University Medical Center Hamburg-Eppendorf, Hamburg, Germany; 2 Department of Radiology and Neuroradiology, University Hospital of Schleswig-Holstein, Kiel, Germany; 3 Department of Anesthesiology and Intensive Care Medicine, University Hospital of Schleswig-Holstein, Kiel, Germany; 4 Clinic of Cardiac Surgery, University Hospital of Schleswig-Holstein, Kiel, Germany; 5 Department of Radiology and Neuroradiology, Molecular Imaging North Competence Center (MOIN CC), University Hospital of Schleswig-Holstein, Kiel, Germany; 6 Department of Mechanical Engineering, Kiel University of Applied Science, Kiel, Germany; 7 Anatomical Institute, University of Kiel, Kiel, Germany; 8 Department of Pathology, University Hospital of Schleswig-Holstein, Kiel, Germany; 9 Institute of Clinical Research and Systems Medicine, Health and Medical University Potsdam, Germany, Potsdam, Germany; 10 Clinic of Vascular Medicine, University Heart and Vascular Center Hamburg, University Medical Center Hamburg-Eppendorf, Hamburg, Germany

**Keywords:** Vascular graft, Bypass, Decellularization, Bioengineering, Vascular surgery, Tissue engineering

## Abstract

The aim of this study was to systematically compare standard detergents for the generation of Decellularization Vascular Grafts (DVG) in terms of their influence on vascular key characteristics. The most common enzymatic and chemical detergents for decellularization were identified from literature, standardized and included: i) Trypsin, ii) Sodium Dodecyl Sulfate (SDS)- and iii) Triton X-100. All protocols were applied to porcine vessels and the manufactured DVG were analyzed for histological, ultrastructural morphology and biomechanical characteristics. Further, DVG were seeded with Human Umbilical Vein Endothelial Cells (HUVEC) and cultured in a bioreactor to investigate biocompatibility after decellularization. Anti-Coagulation properties were assessed by the Chandler Loop model and a platelet-activation–assay. The Trypsin and SDS treatment were the most effective protocols in terms of tissue clearance but both impaired the ultrastructural integrity of the vessel wall in contrast to the Triton X-100 treatment. Moreover, biomechanical characteristics in the test stand did not differ significantly across the applied protocols but treatment of DVG with Trypsin was associated with a reduced Young’s modulus and injuries in the vessel wall in a pulsatil flow model after 30 d. Moreover, coagulation was decreased in the Trypsin-treated group and was slightly increased in the SDS group but no significant difference towards the control group was noted. DVG after Triton X-100 treatment were the only ones capable for successful cell seeding. The here presented experimental data emphasized the main advantages and disadvantages of the most common enzymatic and chemical detergents for the manufacturing of DVG.

**Graphical Abstract:**

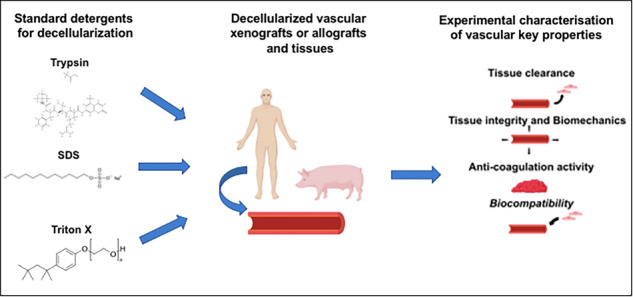

## Introduction

Decellularization of tissues and/or whole organs is a routinely used method for the creation of scaffolds and grafts in tissue engineering. Especially, the decellularization of allogeneic or xenogeneic vessels is a common strategy in vascular tissue engineering [1–3]. Hence, various procedures ranging from biochemical to physical decellularization protocols have been established during the last three decades. Most of these multi-step protocols include several standard detergents for enzymatic and/or chemical decellularization which are usually combined with physical applications e.g., high-pressure-treatments or cryopreservation [1–4]. However, relatively less is known about the potential influence of these detergents on biological and biomechanical characteristics of small and/or medium sized vessels and the generated Decellularized Vascular Grafts (DVG) [1–5]. Especially the preservation of vascular key characteristics like vessel wall integrity, elasticity, tensile strength, cell seeding, biocompatibility and anti-coagulation properties are crucial for the long-term performance of DVG and/or whole organ replacement. Until now, a significant impact of these routinely applied decellularization detergents on vascular key features could neither be precluded nor has been systematically investigated in detail [1–5]. It has also been hypothesized that the chemical and enzymatic decellularization step is potentially the most critical for tissue integrity [4–6]. Gilbert (2012) have already emphasized that the literature is full of conflicting results in terms of decellularization procedures and systematic and standardized studies are needed to determine the effects of various standard detergents on each particular tissue product of interest [5].

Accordingly, the primary objective of this study was to systematically compare these standard detergents in terms of their influence on vascular key characteristics of DVG.

Therefore, the current experimental study identified and encompassed the most frequently used enzymatic and chemical decellularization procedures of DVG from original and major reviewing articles including (i) Trypsin, (ii) Sodium Dodecyl Sulfate (SDS)- and (iii) Triton X-100 lysis buffer-based protocols [2, 3, 6–16]. If several variants in terms of detergent concentrations and exposure times were described, the most tissue preserving protocol was initially selected in order to standardize the methodology.

## Material and methods

### Ethics

Protocols were approved by the local ethics committee of the University Medical Center Schleswig-Holstein, Kiel, Germany (protocol identification numbers: D513/19, D518/13 and D451/21). All procedures were performed in accordance with the Helsinki Declaration of 2013.

### Preparation of the decellularized vascular grafts

Porcine tissues are the most common xenogeneic scaffolds for cardiovascular tissue engineering and therefore used in the current study [1–3, 5, 7]. The whole aorta of German Landrace pigs (*n* = 10) was obtained from a local slaughterhouse in Schleswig-Holstein. Initially, the aorta was explanted and transported in PBS/EDTA and Penicillin G -Streptomycin (1 mM) at 4 °C. Surgical preparation of standardized grafts with a length of 60 mm ± 2.17 mm, a diameter of 22 mm ± 2.23 mm from the infrarenal aortic segment was performed after arrival in the laboratory and afterwards the respective decellularization protocols were performed. The generated scaffolds were used as an intact DVG or to prepare homogenized vascular extracellular matrix.

### Protocols for the generation of decellularized vascular grafts (DVG)

Prior to the experiments, established and routinely performed decellularization protocols were identified and extracted from major reviewing and original articles regarding “decellularization”, “tissue engineering” and “(cardio)vascular grafts” [2, 3, 6–16]. Three standard chemical and enzymatic detergents and respective basic protocols for decellularization of vascular grafts were identified and included in the present study: biochemical decellularization was performed with (1) Trypsin (BioWest, Nuaillé, France) (2) Sodium Dodecyl Sulfate (SDS) (Carl Roth, Karlsruhe, Germany) and (3) Triton® X-100 (Carl Roth, Karlsruhe, Germany) lysis buffer-based protocols. Phosphate Buffered Saline (PBS-/-) served as control group as summarized in Table 1. All protocols were performed in accordance with the identified literature. If several variants in terms of detergent concentrations and exposure times were described, the most tissue preserving protocol was initially selected in order to standardize the methodology. Briefly, after preparation of the porcine aortas, the tissue samples were treated in three consecutive steps: firstly, tissues were either treated with 0.1% Trypsin + 0.04% EDTA/PBS-/- for 24 h or with 0.1% SDS/PBS-/- or 0.25% or 1% Triton X-100 + 0.2% EDTA/PBS-/- or only PBS-/- as control for 48 h or 72 h respectively (Table 1). To remove detergents and cellular debris prior to enzymatic treatment, samples were intensively washed after 24 h and 48 h, respectively, 3 times with deionized water and 3 times with EDTA/PBS-/-. Secondly, all protocols were further digested with 150 mg/mL RNase and 150 IU/mL DNase for 5 h at 37 °C, washed 10 times in EDTA/PBS-/- and stored at 4 °C as summarized in Table 1.

**Table 1:** Overview of the established decellularization protocols from the literature

Trypsin	SDS	Triton X-100	PBS/Control
0.1% Trypsin	0.1% SDS	0.25% Triton X-100^a^	PBS -/-
0.04% EDTA	PBS -/-	0.2% EDTA	
PBS -/-		PBS -/-	
24 h	48 h	48 h^b^	48 h
All decellularization procedures were concluded with Ribonuclease (RNase 150 mg/mL) Deoxyribonuclease (DNase 150 IU/mL) 5 h at 37 °C and washed 10 times with EDTA/PBS-/-			

^a^Decellularization with 1% Triton X-100 was additionally performed

^b^Decellularization for 72 h was additionally performed for 0.25% and 1% Triton X-100

### DNA Preparation and quantification of DVG

The decellularized tissues were homogenized after placing them in 1X PBS with the tissue homogenizer for 5 min on ice. Afterwards, 100 mg tissues were transferred into 1 ml Guanidinium Thiocyanate solution (TRizol solution; Invitrogen, Carlsbad, USA) and stored overnight at 4 °C. After centrifugation with 12,000 × *g* for 5 min at 4 °C, the supernatants were mixed with 200 µl chloroform and centrifuged again at 12,000 × *g* for 5 min at 4 °C. The resulting RNA-containing supernatants were discarded. The DNA-containing solution was mixed with 300 µl 100% ethanol and centrifuged at 2000 x g for 5 min at 4 °C. Supernatants were discarded again and pellets were then resuspended in 300 µl 8 mM NaOH and centrifuged at 2000 × *g* for 5 min at 4 °C. Pellets were again resuspended with 300 µl 75% ethanol and centrifugated at 2000 × *g* for 5 min at 4 °C. DNA-containing pellets were air-dried for 10 min and resuspended with 300 µl 8 mM NaOH. After final centrifugation at 12,000 × *g* for 10 min at 4 °C, supernatants were buffered with 0.1 M HEPES (Thermo Fisher, Waltham, USA). All specimens were dissolved in 100 µL DNase-free water and analyzed with the NanoDrop 2000C (Thermo Fisher, Waltham, USA). Absorbance at 260 nm were used to obtain the DNA concentration from a standard curve and the ratio 260 nm/280 nm to evaluate the purity of the samples. The ng of DNA per mg of dry weight of the tissue is calculated.

### Histological analysis of the DVG

DVG were fixed in 4% formalin and embedded in paraffin. Afterwards, the DVG were divided into a proximal, middle, and distal section of 2 cm each. From each section, 3 samples, 4 μm thick each, were prepared and routinely stained with Haematoxylin-Eosin (HE) and Elastica van Gieson (EvG). For evaluation of cell nuclei, six High Power Fields (HPF) were randomly counted by two independent observers and cell nuclei were determined as mean of all evaluated HPF.

### Analysis of ultrastructure of the DVG by scanning electron microscopy (SEM)

Ultrastructure of the DVG were analyzed by Scanning Electron Microscopy (SEM). Samples were chosen from the proximal, middle and distal section of each DVG similar to the histological analysis. Vessel specimen were fixed in 2.5% glutaraldehyde and postfixed in 2% osmium tetroxide, irrigated with distilled water and then dehydrated in series of 50–100% ethanol. They were then placed in 100% ethanol and dried by critical point dryer (CPD030, Bal-Tec AG, Balzers, Lichtenstein). The specimens were mounted onto SEM slabs and sputter-coated for 50 seconds, 38 mA with gold by sputtercoater (SCD050, Bal-Tec AG). Examination of the vessel specimens was performed with a JSM-IT200 (JEOL, Tokyo, Japan).

### Tensile and suture strength of the DVG

Tensile and suture strength characteristics of the grafts were evaluated in a standard tensile test station (Z 0.5 Zwick/Roell, Ulm, Germany) as reported before [17]. Briefly, DVG were mounted on an irregular polymer surface to prevent slippage during the analysis (Fixation zone of each graft: 5 mm). After applying the mechanical force at a uniform strain rate (1 mm/min), the DVG ruptured near the middle or near both ends. Force measurements was recorded by auditing software testXpert II (Zwick/Roell). For tensile strength testing, a standard synthetic, non-absorbable, monofilament surgical suture (Prolene monofil FS2 5-0, Ethicon, Cincinnati, USA) was placed 3 mm towards both edges of the vascular graft.

### Shelf-life analysis of DVG in a pulsatil flow model

Durability and shelf-life of the decellularized vascular grafts were assessed by implantation into an established, slightly modified pulsatil flow model [18, 19]. Briefly, the grafts were fixed in a customized flow chamber which was filled with a solution of 0.90% w/v of NaCl and perfused for 30 d. The solution of the whole circuit was exchanged once a week to avoid fungi and bacterial contamination. A pulsatile pump (Cardiovascular Waveform Pump; Harvard Apparatus, Holliston, USA) provided physiological flow conditions with a stroke volume between 70 and 100 ml and a systolic pressure between 90–150 mmHg and a diastolic pressure between 35 and 70 mmHg.

After 30 d, the tissues were systematically examined by two independent senior pathologists, using a quadrant system. Aim of this investigation was the identification of macroscopic injuries e.g., tears of the extracellular matrix and/or complete dissections of layers. Further, all samples were scanned microscopically in order to identify non-visible disruptions of the matrix. In addition, the fluid of the circuit was routinely checked for tissue-related emboli.

### Cell sources and culture for cell seeding of DVG

Human Umbilical Vein Endothelial Cells (HUVEC) were isolated from umbilical cords as described before and cultured in endothelial cell growth medium ECGM (PromoCell, Heidelberg, Germany) supplemented with 4 μL/mL of endothelial cell growth, 0.1 ng/mL epidermal growth factor, 1 ng/mL basic fibroblast growth factor, 90 μg/mL heparin, 1 μg/mL hydrocortisone (all from PromoCell) and 10% fetal bovine serum (Thermo Fisher, Waltham, USA) [20].

### Cell viability after cell seeding of DVG

To assess biocompatibility, HUVEC were seeded on the surface of the DVG in a rotating bioreactor (Aptus Bioreactors, Clemson, USA) for 5 d [17]. The bioreactor settings were chosen by experimental pre-testing to enable a continuous flow during reactor movement, regular rest periods for cell seeding and prevention of exceeded wall-attachment. After a period of static immersion in HUVEC culture medium for 4 h, the bioreactor was rotated at 40° and 60°, respectively and at 1 rotation per minute. There was a programmed rest period for 10 min after each cycle. An air pump was attached to the rotator jars via a sterile inline filter, continuously enriching the culture media with air from the CO_2_ incubator. Cultivation was performed under humidified conditions at 37 °C and 5% CO_2_.

After 5 d, seeded HUVEC were qualitatively assessed by fluorometric visualization. Fresh medium containing 0.1 µM Calcein AM (Thermo Fisher) and 0.2 ng/ml Hoechst 33342 (Invitrogen, Carlsbad, USA) were added to the grafts for the assessment of cell viability and cell nuclei, respectively. Cells were stained for 30 min inside the dark incubator.

### Live-3D-cell-imaging of seeded cells on DVG

Samples of the vascular grafts were visualized on Leica THUNDER Imager 3D assay (Leica Mikrosystems GmbH, Wetzlar, Germany) based on Leica DMi8 microscope with 5x objective lens (PLAN 5x/0.12 dry), 20x objective lens (HC PL APO 20x /0.80 dry) and Leica K5 camera (4.2 MP, 40 FPS) as described before [17]. Briefly, cool LED pE4000 was used as a fluorescence light source and the filter cubes with the desired excitation and emissions were selected (DAPI or LED-405 EX: 405/60, EM: 470/40; or FITC EX:480/40, EM:527/30 or TXR EX:560/40, EM:630/75). Normal or top to bottom Z-stack images of the samples were acquired using the same set of filters and settings (intensity, exposure time, threshold and Z-step size). Images were processed with Leica LAS X software and the small volume computational clearing was applied. Immunofluorescence staining was analyzed using the ImageJ software 1.41 (National Institutes of Health NIH). Cell numbers and densities (cell number/cm^2^) were evaluated in fluorescent images using the automatic image-based tool for counting nuclei from the Image J software 1.41 (NIH).

### Anti-coagulation activity of DVG in the Chandler Loop model

To analyze anti-coagulation properties of DVG, the established Chandler Loop system was used as previously described [21, 22]. Briefly, DVG were fixed in silicon tubes and filled with whole blood from three donors who underwent full coagulation diagnostics prior to the experiments. The DVG then rotated in a constantly warmed water bath at 37 °C and maximal clotting formation within the lumen of the vascular grafts was assessed by weighing and measuring the thrombus after 30 and 60 min, respectively.

### Platelet-activation–assay

The activation of platelets by the surface of the various DVG was assessed by a standard platelet-activation–assay. Briefly, Platelet-Rich Plasma (PRP) was obtained from three donors who underwent full coagulation diagnostics prior to the experiments.

Pieces of DVG (2 × 2 mm) were fixed on the bottom of a 12 well plate. A solution of 50% citrated blood and CaCl_2_ (0.002 M) was prepared, vortexed for 20 s, and centrifugated at 1800 × *g* for 10 min. The obtained blood plasma was again centrifugated four times at 1800 × *g* to receive PRP. Then, 200 µL of PRP were placed into each well. The control group was composed of PRP only and therefore without surface activation of thrombocytes by the DVG. After 30 min, the wells were washed with PBS solution to stop clotting and remove soluble clots. Finally, the total number of clots was visually assessed via microscopic examination.

### Statistical analysis

Each experiment was repeated three times if not stated otherwise. Data were analyzed by using Graph Pad Prism version 9.2.0 (Graph Pad Software; San Diego, USA). All data are presented by the Mean ± Standard Deviation (SD). Categorical variables are presented as frequency distributions (n). Normality was tested by using the Shapiro-Wilk and Kolmogorov-Smirnov test (alpha = 0.05). Data were analyzed using one-way ANOVA or Kruskal-Wallis-Test, if appropriate. Tukey’s or Dunn’s Multiple Comparison was performed as Post-hoc-test. A *p* value < 0.05 was considered as significant.

## Results

### Histological and ultrastructural examination of DVG

**Fig. 1:**
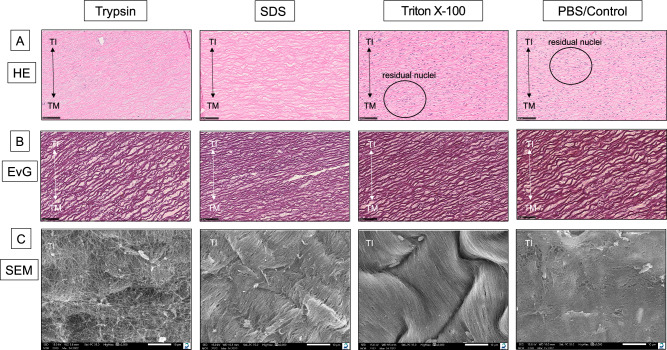
**A**, **B** Representative depiction of histological staining of the Decellularized Vascular Grafts (DVG) after treatment with Trypsin, SDS, Triton X-100 or PBS/Control with Haematoxylin and Eosin (HE) and Elastica-van-Gieson (EvG). **C** Scanning Electron Microscopy (SEM) of the DVG. TI = Tunica Intima; TM = Tunica Media; Triton X = 0.25% Triton X-100 (48 h); magnification HE: ×200; EvG: ×200; SEM: ×2000

Visual examination of HE and EvG-stained tissues after decellularization-procedures revealed an intact Tunica media and the absence of a Tunica intima due to complete removal of endothelial cells in the inner lumen (Fig. 1A, B). The thickness of the preserved Tunica media did not differ significantly between the three applied protocols (data not shown) but a relevant higher amount of residual cell nuclei could be found after Triton X-100 treatment (Fig. 1A). These findings were also supported by SEM of all specimens: ultrastructural analysis revealed remaining cell debris in Triton X-100 and PBS/Control-treated tissue samples. However, surface and vessel wall integrity were only completely preserved in the Triton X-100-treated group whereas the SDS and Trypsin-treated tissues showed a rough surface and impairments of the ultrastructural composition (Fig. 1C).

### Quantification of decellularization of DVG

**Fig. 2:**
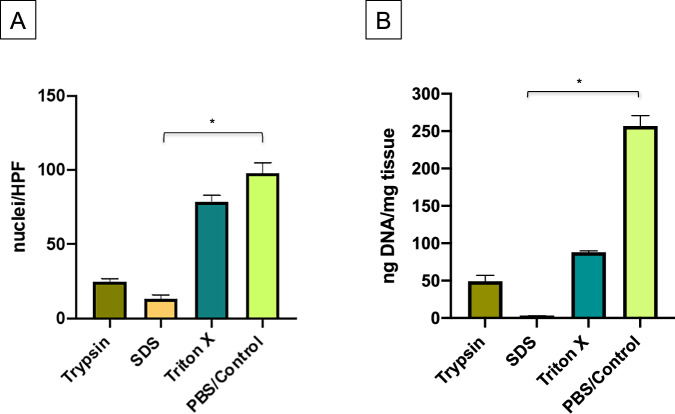
**A** Quantification of cell nuclei per High Power Field (HPF). **B** DNA content represented as ng DNA per mg dry tissue after decellularization. Triton X = 0.25% Triton X-100 (48 h); Kruskal-Wallis- and Dunn’s Multiple Comparison Test; * = *p* < 0.05

The assessment of efficacy of decellularization methods was based on (1) histological staining of cell nuclei and (2) quantification of DNA from tissue samples as reported before by Crapo and colleagues [5, 8]. A significant lower number of cell nuclei per HPF was found in tissue samples treated with SDS (SDS: 13.33 ± 2.52 vs PBS/Control: 98 ± 7 nuclei/HPF; *p* < 0.05) whereas the specimens treated with the Trypsin or with Triton X-100 protocol did not differ significantly from the PBS/Control group, as presented in Fig. 2A. Corresponding results were determined by DNA quantification of tissue samples ranging from significant lower values in the SDS group (SDS: 2.77 ± 0.40 ng DNA/mg tissue vs PBS/Control: 257 ± 14 ng DNA/mg tissue; *p* < 0.05) to sufficient reduced DNA measurements in the Trypsin (49.24 ± 7.96 ng DNA/mg tissue) and strong decreased DNA values in the Triton X-100 group (87.95 ± 2.00 ng DNA/mg tissue) compared to the PBS/Control group. Overall, both the Trypsin and SDS protocol fulfill the objective criteria of sufficient decellularization but only statistical comparison between the SDS and the PBS/Control group was significant (*p* < 0.05) (Fig. 2B).

In addition to the initial experiments, two further Triton X-100 protocols were conducted due to the fact that the initially identified protocol could not reach the threshold for complete decellularization. Both, the increase to 1% Triton X-100 and the extension of exposition (72 h) for the 0.25% and 1% Triton X-100 protocol resulted in sufficient decellularization (Supplementary 1).

### Biomechanical analysis of DVG

**Fig. 3:**
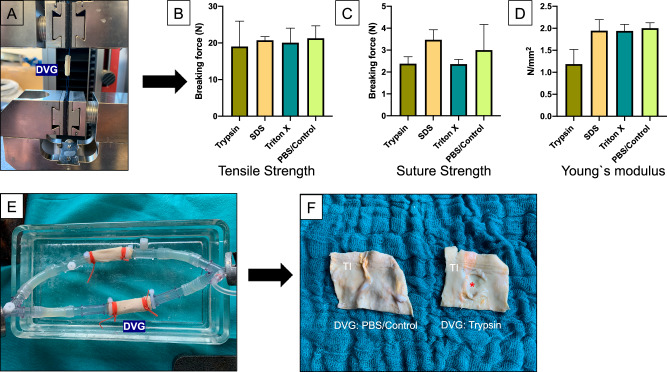
**A** Biomechanical characteristics of the Decellularized Vascular Grafts (DVG) were analyzed in a tensile test station: **B**–**D** Tensile strength, suture strength and Young’s Modulus of the DVG were assessed. **E** Shelf-life of the respective grafts were evaluated in a pulsatil bioreactor for 30 d. **F** Macroscopic injuries/dissections (red asterisk) of the vascular Tunica Intima (TI) were only observed in the Trypsin-treated group after 30 d of continuous circulation. Triton X = Triton 0.25% X-100 (48 h)

To evaluate the possibly altered mechanical characteristics of the decellularized vascular grafts, tensile and suture strength as well as Young’s modulus was evaluated in the tensile test station (Fig. 3A).

Tensile and suture strength did not differ significantly between the Trypsin, SDS, Triton X-100, and PBS/Control group (Fig. 3A, B). Also, the Young’s modulus did not vary significantly across the groups (Fig. 3D). However, DVG after Trypsin-treatment gained the lowest biomechanical test values compared to SDS and Triton X-100- treated DVG and especially, the Young’s modulus was notably lower (Fig. 3D). These findings also correlate with the results from a circulation model which was used for in vitro shelf-life and durability testing of DVG under physiological conditions: macroscopic injuries were observed in 2 out of 3 Trypsin-treated grafts but no tissue-related emboli were observed in the fluids across all groups (Fig. 3E). Beyond the observed macroscopic injuries in the Trypsin group, no further macroscopic or histological impairments were examined in the SDS, Triton X-100 and PBS/Control group (Fig. 3F).

### Cell seeding and biocompatibility of DVG

**Fig. 4:**
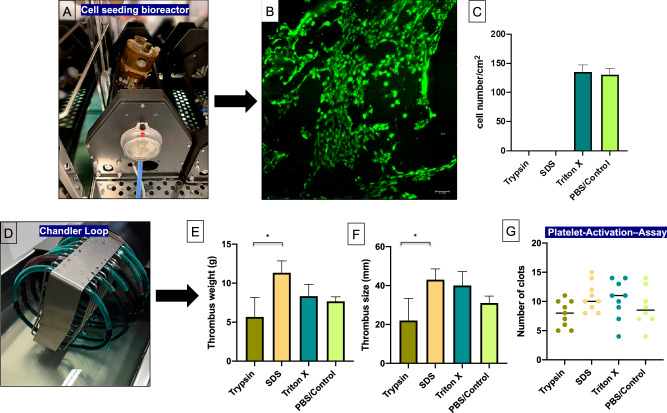
**A** Cell seeding of the Decellularized Vascular Grafts (DVG) was performed in a rotating bioreactor (Aptus Bioreactors, Clemson, USA). **B**, **C** Cell seeding was qualitatively and quantitatively assessed by fluorometric Live-3D-Cell-Imaging after 5 d. **D** The Chandler Loop model was applied for the analysis of coagulation activity of the DVG. **E**, **F** Thrombus weight (g) and thrombus size (mm) after 30 and 60 min were assessed. **G** A platelet-activation–assay was performed to test the activation of platelets by the surface of the DVG. Scale bar: 10 µm; Triton X = Triton 0.25% X-100 (48); Kruskal-Wallis- and Dunn’s Multiple Comparison Test; * = *p* < 0.05

Various approaches in vascular tissue engineering have been rooted in reendothelization of the decellularized surfaces of the vascular grafts. Therefore, biocompatibility of the different decellularization protocols have been investigated by seeding HUVEC on the surface of the various vascular grafts (Fig. 4A–C). Successful experimental cell seeding was only observed in the Triton X-100 and the PBS/Control group whereas the Trypsin and SDS group did not show any metabolic active cell layer after cell seeding and cultivation for 5 d (Fig. 4C). Even the increase (1%) and extension of exposure time (72 h) of the Triton X-100 protocol demonstrated a sufficient cell seeding compared to the initial protocol and the PBS/Control group (Supplementary 1).

### Anti-coagulation characteristics of DVG

Preserved anti-coagulation activity of the DVG surface were assessed by the Chandler Loop model (Fig. 4D). After 30 min no thrombotic material was observed in any of the experimental groups (data not shown). After 60 min thrombus formation occurred in each graft and in all four groups (Fig. 4E, F). However, only thrombus weight (5.67 ± 2.52 g) and size (22 ± 11.36 mm) of Trypsin-treated vascular grafts showed significant accelerated anti-coagulation activity in contrast to the SDS-treated grafts (thrombus weight: 11.33 ± 1.53 g; thrombus size: 43 ± 5.57 mm) (*p* < 0.05). In comparison, vascular grafts after Triton X-100 treatment demonstrated comparable anti-coagulation activity towards the PBS/Control group (Fig. 4E, F). Moreover, the platelet-activation–assay demonstrated that no significant increased platelet clots on the surface of the various DVG was observed compared to the control group (Fig. 4G).

## Discussion

**Fig. 5:**
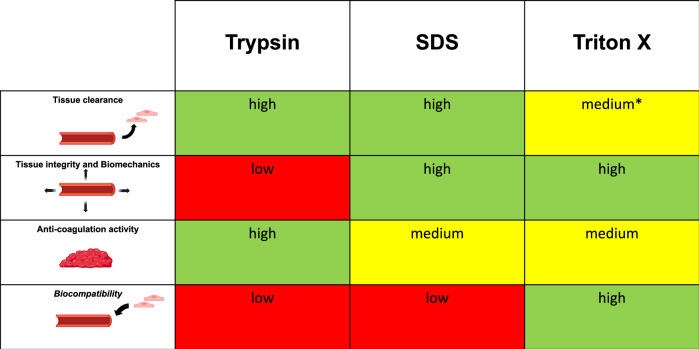
Overview of the impact of the Trypsin, SDS, and Triton X-100 protocols on the key characteristics of the Decellularized Vascular Grafts (DVG) relative to the PBS/Control group. *Graded “medium” because tissue clearance was not fully complete with the initial described 0.25% Triton X-100 protocol but fully sufficient with the additional Triton X-100 protocols as described in Table 1

The current study compared systematically the three most frequent enzymatic and chemical detergents and respective protocols for the decellularization of vascular grafts. All protocols are commonly used in multistep approaches combined especially with physical methods. However, the chemical and enzymatic decellularization substep is the most critical for the vascular tissue integrity and its biomechanical properties but also the less investigated in vascular tissue engineering weight [1–3, 5]. The current experiments revealed that Trypsin and SDS protocols were the most effective protocols in terms of tissue clearance, but the ultrastructural integrity of the vessel wall was impaired in both groups. In contrast, the Triton X-100 protocol was less effective for decellularization but most tissue preserving. However, additional extension and increase of the Triton X-100 protocol demonstrated a similar potency of decellularization compared to Trypsin and SDS. Moreover, biomechanics in the test stand did not differ across the applied protocols but treatment with Trypsin was associated with reduced Young’s modulus and increased vascular injuries in the pulsatil flow model after 30 d. Moreover, DVG after Triton X-100-decellularization were the only grafts showing tendency of cell seeding – even after the application of increased and extended protocols. Anti-coagulation activity was accelerated in the Trypsin-treated group and slightly increased after treatment with the SDS but no significant difference compared to the control group was noted. An overview of all major findings is given in Fig. 5.

### Efficacy of tissue decellularization

Objective evaluation of the efficacy of decellularization have been described by Crapo et al. 2011 including the absence of nuclei per HE-staining, and less than 50 ng DNA per mg dry tissue weight [5, 8]. Based on these criteria, only the Trypsin and SDS protocol generated sufficient nuclei- and DNA-free tissues as a singular decellularization step. In contrast, Triton X-100 was less effective with multiple residual cell nuclei and a relatively high amount of DNA within the extracellular matrix. Therefore, various authors have already prolonged the exposure time of Triton X-100 treatment to 72 h but it was mostly combined with a second and/or third decellularization treatment [6, 9]. However, both additionally applied Triton X-100 protocols with increase to 1% and prolonged exposure time of 72 h confirmed that Triton X-100 can also lead to complete and effective decellularization as a standalone decellularization step. Consequently, the exposure time of Triton X-100 must be extended, or the particular protocol must be combined with other e.g. physical and/or decellularization methods in order to gain sufficient vascular tissue clearing. Therefore, the 0.25% Triton X-100 treatment for 48 h only appears sufficient as a substep of a multistep protocol in combination with other physical and/or chemical decellularization methods.

Finally, it is worth to mention that not all vascular tissue engineering strategies are based on the preservation of the distinct vessel structure. An increasing number of approaches focus on the manufacturing of bio-inks for 3D bioprinting based on decellularized tissues. In this context, the use of tissue-aggressive but also highly effective protocols such as trypsin might be preferred [5, 23].

### Tissue integrity and biomechanics of DVG

Histological and ultrastructural analysis revealed that only the Trypsin and SDS protocol enabled sufficient tissue clearance with absence of remaining cell nuclei and debris but at the cost of integrity of the vessel wall. Trypsin as a serine protease and SDS, as an anionic detergent, are known to disrupt cell-matrix interaction and their detrimental effects especially on collagen (Trypsin) and glycosaminoglycans (SDS) have been reported previously [1–3, 5, 24, 25]. Accordingly, the relatively low elasticity (Young’s modulus), suture strength and the occurrence of macroscopic injuries in the Trypsin-treated vascular grafts were apparently associated with ultrastructural impairments of the remaining Tunica media [2, 3, 5, 10, 13, 26]. Hence, it has also been demonstrated that both concentration and treatment time of Trypsin and SDS- based protocols could lead to significant structural alterations of the vessel wall and biomechanical characteristics and their application should be avoided in small vessels or restricted to a minimum in general [2, 3, 5, 6, 10, 13, 26, 27]. However, several authors also emphasized that Trypsin can be a pivotal decellularization step in thick, porcine cardiovascular tissues [5, 24, 27, 28].

In contrast, the Triton X-100 treatment was more tissue preserving and enabled an excellent biomechanical performance of the decellularized vascular grafts. This nonionic surfactant has a disruptive effect on the polar head group and hydrogen bonds within the lipid membrane [1–3, 5]. Thus, Triton X-100 treatment has been reported in various cardiovascular tissue engineering approaches especially in combination with low-dose ionic detergents and physical methods of decellularization [5, 6, 14, 16]. These strategies are mainly based on the relatively gently exposure and dissolvement of cell nuclei from the extracellular matrix by Triton and the high efficacy of a second, more tissue-aggressive protocol.

### Biocompatibility of DVG

Commonly, most tissue engineering approaches using decellularized cardiovascular grafts are conceptualized for cell seeding or reendothelialization either ex vivo in a bioreactor or in vivo in a host organism [1–3, 24, 29]. Accordingly, endothelia cell seeding on the decellularized matrix is mandatory for cardiovascular tissue engineering. Interestingly, only Triton X-100 treatment allowed consecutive cell seeding in the current experiments although all tissues have been treated carefully with multiple washing steps. Various authors have already reported residues of chemical detergents within the extracellular matrix which could lead to a disruptive effect on cell seeding and integration [5, 30]. Especially SDS tends to remain in treated tissues and its basement membrane complex contributes to an altered structure and cell signaling of the extracellular matrix of DVG [5, 24, 30, 31]. This might have limited the confluence of seeded endothelial cells. Other authors had therefore proposed a secondary rinsing with Triton X-100 as a possible way to reduce the SDS content in decellularized tissues [5, 6]. This general problem of an effective remodeling of DVG could also be addressed by bioengineering e.g. by surface modification of DVG with antibody related “capture mechanisms” which bind to circulating endothelial cells [1, 4, 30].

### Anti-coagulation activity of DVG

Another pivotal benchmark parameter for successful vascular bioengineering is ensuring that the grafted vessel has a sufficient anticoagulant barrier of the grafted vessel and anti-platelet-activity. However, this aspect is relatively less investigated within vascular bioengineering and most authors have only reported limited in vitro experimental and just mid-term animal data so far [1–4, 24, 30].

Interestingly, the Trypsin-treated DVG showed relatively low thrombus formation and platelet aggregates during the experiments compared to the PBS/Control group. Accordingly, several investigations have strengthened the observation that there is a narrow zone of trypsin concentration within which blood coagulation is accelerated, whereas in higher concentrations coagulation is inhibited [32–34]. In order to exclude a bias due to residual detergents, activity assays for trypsin, SDS, and Triton X-100 were additionally performed which showed practically no or minimal residues of the detergents after the washing processes (Supplementary 2). It can therefore be speculated that the use of Trypsin may lead to structural changes in the extracellular matrix of the DVG, which causes an altered activation of the coagulation cascade in contrast to native vessels. Further research appears necessary in this context. In contrast, Triton X-100-treated DVG showed similar anti-coagulation activity compared to the PBS/Control group whereas SDS-treated grafts were prone to thrombus formation in the Chandler Loop. Although no SDS activity was detected in the current samples, molecular residues could have influenced these results and should be taken into account when using SDS for decellularization (Supplementary 2) [5, 24, 30]. It has been demonstrated that molecular, barely detectable residues of SDS within the extracellular matrix can induce additional coagulation of platelets and fibrin [11, 24]. A previous study confirmed that anionic compounds such as polyphosphate and SDS are able to accelerate plasmin- and Thrombin-Activatable Fibrinolysis Inhibitor (TAFI) activation - even in very small amounts [35]. Accordingly, the present findings underline the necessity to develop advanced assays and tests that can accurately detect the presence of chemicals used in this study, preferably in a quantitative fashion. Additional surface coating and/or biological modification of tissue engineered vascular grafts could further improve anti-coagulation properties and compensate disadvantages of decellularization treatments associated with residual chemicals and exposure of extracellular matrix components [4, 30].

## Conclusion

The presented experimental data emphasize the main advantages and disadvantages of basic enzymatic and chemical detergents for the decellularization of vascular grafts. Trypsin and SDS were identified as strong detergents producing sufficient DVG even without further processing steps. The initially applied Triton X protocol, on the other hand, did not enable sufficient decellularization at first. However, further extension and/or enhancement of Triton X protocols produced adequate DVG. Triton X-100 preserved all structural properties of the porcine vessels and therefore appear to be very suitable in combination with physical processing steps in a multi-step protocol. The biomechanics of the DVG were particularly impaired by Trypsin, whereas strong coagulation activity was demonstrated with the SDS protocol. Finally, only the Triton X-100 protocols achieved sufficient results in terms of cell seeding of the DVG.

## Supplementary information

Supplement 1

Supplement 2

## Data Availability

The datasets generated during and/or analyzed during the current study are available from the corresponding author on reasonable request.

## Abbreviations

CMC: Chemical Manufacturing Control
d: day(s)
DVG: Decellularized Vascular Graft(s)
EDTA: Ethylenediaminetetraacetic acid
EvG: Elastica-van-Gieson
HE: Haematoxylin and Eosin
HPF: High Power Field(s)
HUVEC: Human Umbilical Vein Endothelial Cell(s)
PBS: Phosphate Buffered Saline
PRP: Platelet-Rich Plasma
SEM: Scanning Electron Microscopy
SDS: Sodium Dodecyl Sulfate
TAFI: Thrombin-Activatable Fibrinolysis Inhibitor
TI: Tunica Intima
TM: Tunica Media
