# Supplementary material for: Systematic analysis of the influence of enzymatic and chemical detergents on structure, biomechanics and biocompatibility of decellularized vascular grafts
Source: J Mater Sci Mater Med. 2025 Nov 26;36(1):114. doi: 10.1007/s10856-025-06967-3 (PMC12669320; doi:10.1007/s10856-025-06967-3)
Supplement: Supplementary file 1 — Supplement 1 [file 10856_2025_6967_MOESM1_ESM.pdf]

Supplement 1

Examples from additional Triton X-100 protocols:

Tissue sample	Protocol	Tissue weight (mg)	Total ng DNA	ng DNA/mg of dry weight of the tissue
Porcine Aorta	0.25 % Triton X-100 (72 h)	109	2816.4	25.8
Porcine Aorta	1 % Triton X-100 (48 h)	70.9	2573.0	36.3
Porcine Aorta	1 % Triton X-100 (72 h)	98.1	3685.6	37.6
Porcine Aorta	PBS (control)	74.3	17871.8	240.5

Cell seeding for the additional Triton X-100 protocols:

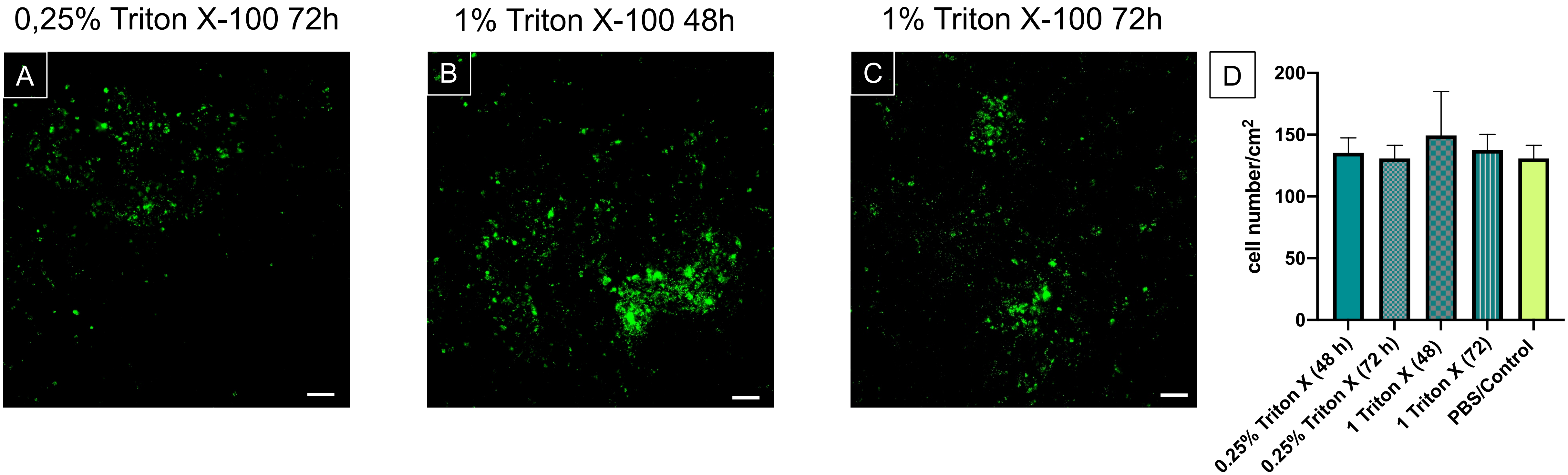

A) – C) Re-seeded HUVEC were maintained in culture and stained with CalceinAM as described in *2.10* in the *Materials and Methods* section. D) No significant difference between the different Triton X protocols was observed in terms of cell seeding (Kruskal-Wallis-Test). Bars denote 50µm.
