# Supplementary material for: Systematic analysis of the influence of enzymatic and chemical detergents on structure, biomechanics and biocompatibility of decellularized vascular grafts
Source: J Mater Sci Mater Med. 2025 Nov 26;36(1):114. doi: 10.1007/s10856-025-06967-3 (PMC12669320; doi:10.1007/s10856-025-06967-3)

Supplement 2

Trypsin activity colorimetric assay kit (Abcam #ab102531):

Procedure:

100mg of decellularized tissue was homogenized with 1ml Assay Buffer 1 on ice in the homogenizer and centrifuged 21000xg for 5 min. The supernatants were analyzed in duplicated according to the manufacturer instructions. The STD curve (R^2 = 0,9997) for the Trypsin is created by reading the absorbances at 405nm of the known amounts (nmol) of the enzyme product (*p*-NA) which is stable for 4h.

Trypsin Activity in the test samples is calculated as: $Trypsin\ Activity = (B\ \Delta Tx\ V) * D$ = nmol/min/ml = mU/ml
Where: B = Amount of *p*-NA from the Standard Curve (nmol) generated by Trypsin during the reaction time ΔT (T2 – T1).
ΔT = reaction time (T2 – T1) (min).
V = Amount of pretreated sample volume added to reaction well (in mL).
D = Sample dilution factor.

Unit Definition: 1 Unit = amount of trypsin that cleaves the substrate, yielding 1.0 µmol of *p*-NA per minute at 25°C.
1 *p*-NA Unit (µmol/min) = 0.615 TAME Unit = 35 BAEE Unit. TAME = *p*-Toluene-sulfonyl-L-Arginine Methyl Ester BAEE = Nα-Benzoyl-L-Arginine Methyl Ester

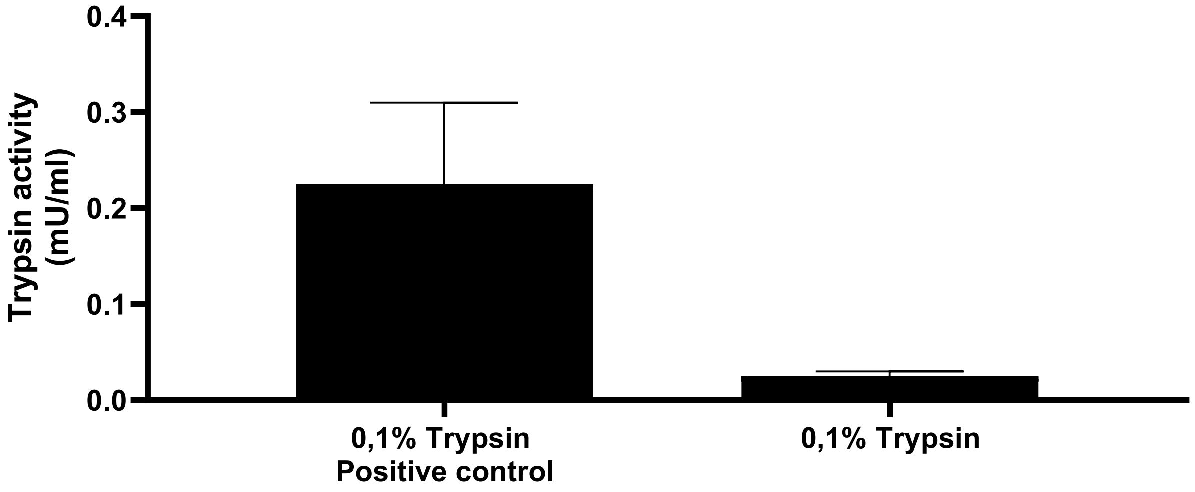

Detergent assay kit (Sigma #MAK459):

Procedure:

100mg of decellularized tissue was homogenized with 1ml of PBS on ice and were centrifuged 21000xg for 5 min.
The supernatants were analyzed in duplicated according to the manufacturer instructions. Absorbances were evaluated at 560nm for Truiton X-100 and at 650 nm for SDS.
For the calculation of the detergent concentration from the absorbances of the samples, standard curves were created for Triton X-100 (R^2=0.9867) and for SDS (R^2=0.9981).

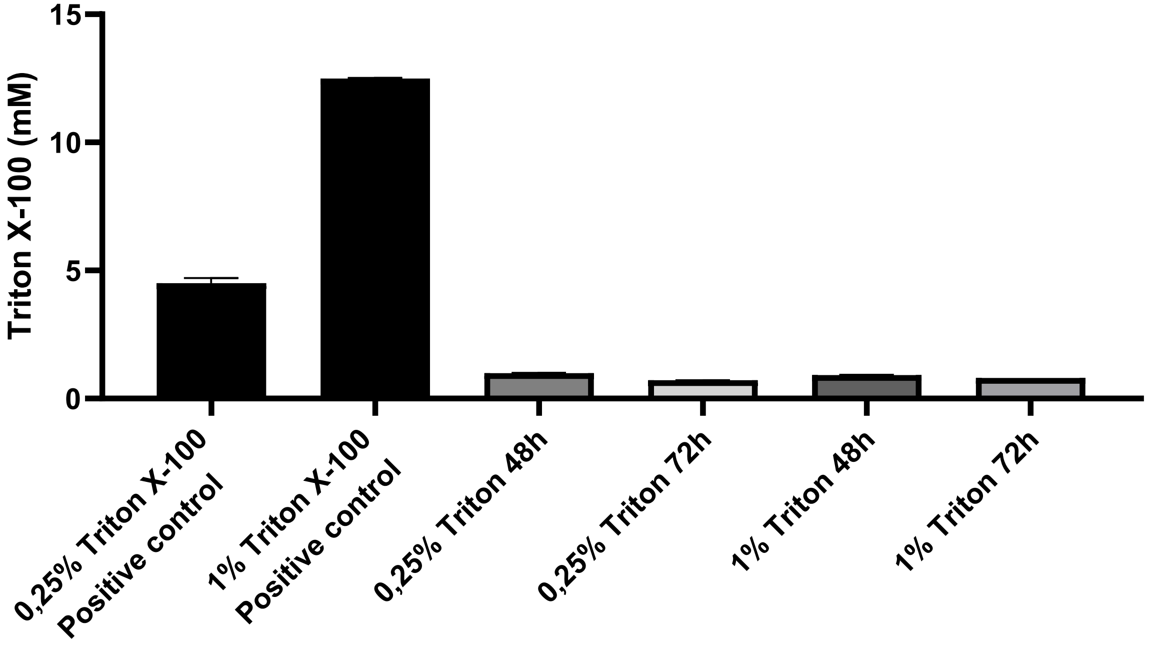

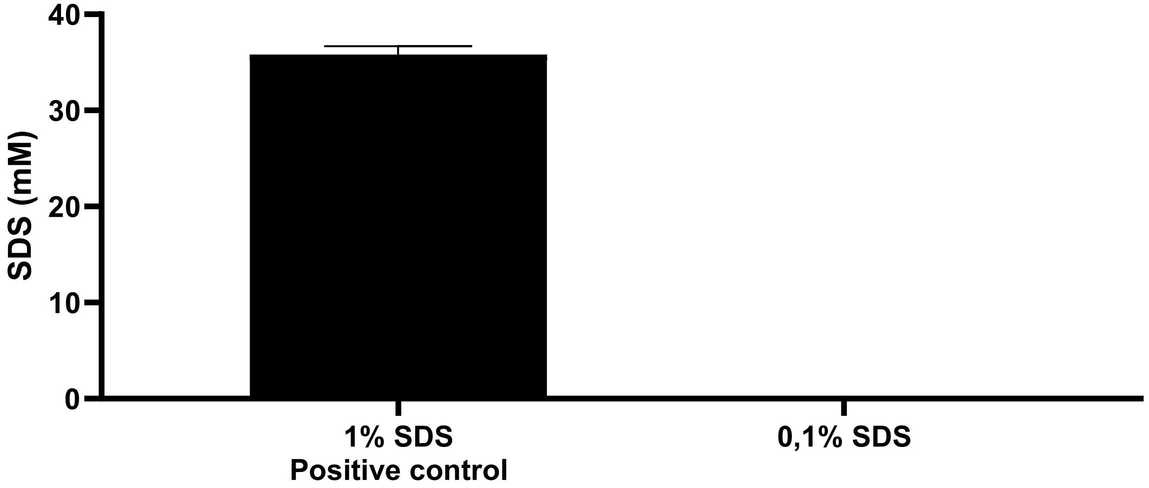

Supplement: Supplementary file 2 — Supplement 2 [file 10856_2025_6967_MOESM2_ESM.pdf]
